# Comparison of Endodontic Retreatment in Teeth Obturated With Resilon or Gutta-Percha: A Review of Literature

**DOI:** 10.7508/iej.2015.04.002

**Published:** 2015

**Authors:** Ciro Soares, Conceição Maia, Fábio Vale, Cícero Gadê-Neto, Lilian Carvalho, Hanieri Oliveira, Rejane Carvalho

**Affiliations:** a *Department of Dentistry, Health School, Potiguar University, Natal, Brazil; *; b *Post-Graduation Program in Odontology, University Potiguar, Laboratory of DentalMaterials, Natal, Brazil*

**Keywords:** Endodontic Retreatment, Gutta-Percha, Resilon

## Abstract

**Introduction:**

Retreatment of endodontically treated teeth is a challenge that requires complete removal of the previous filling material. Several techniques are indicated for this procedure. The present review tries to identify the most efficient method for extirpation of Resilon (RS) root fillings and to compare the speed and efficacy of RS and gutta-percha (GP) root filling removal.

**Methods and Materials:**

Three trained evaluators conducted a search through three major databases (PubMed, Cochrane Library and Lilacs) over the articles published in the period from 2001 to 2014. The search keywords were Epiphany Sealer, Resilon, Retreatment and Removal Procedure.

**Results:**

Twelve articles were included in the final sample (three *in vitro* studies and nine randomized trials).

**Conclusion:**

The ProTaper (manual or rotatory) system in combination with chemical solvents is the most efficient method for removing Resilon root filling. Retreatment of Resilon is more rapid and associated with less remnants of debris.

## Introduction

Despite the development of new technologies and materials, failures are common in endodontic treatment [1, 2]. They usually represent as radiographic changes in periapical tissues and persistent/secondary intra-radicular infection indicating the need for re-intervention [3-6]. 

Persistent bacterial infection in the root canal and periradicular area before and after treatment is the primary cause of treatment failure in endodontic treatment [7]. The first therapeutic option in such cases is endodontic retreatment, for which the complete removal of root filling material is necessary. The main objective of nonsurgical endodontic retreatment is reestablishment of healthy periapical tissues [8]. Different techniques have been indicated for this purpose, including hand and rotatory instrumentation combined with laser and paper point, chemicals, heat or solvents, ultrasonic instruments either in combination or alone [9, 10]. 

Gutta-percha (GP) is the most commonly used root canal filling material composed of zinc oxide and gutta-percha [11], which exhibits properties such as biocompatibility, dimensional stability and ease of removal [12]. However, it does not adhere to any type of sealers [13]. Resilon (RS) (Pentron Clinical Technologies, Wallingford, CT, USA) system is composed of a dual-cure synthetic bio-based polymer cement. This polyester-based resin is used to make cones that are also used in root canal obturation and it has the manipulation properties similar to GP [14]. According to the manufacturer, forming a monoblock within the canal is the ultimate goal of this system [15]. Despite the satisfactory physicochemical results and good compatibility confirmed through intra-osseous and subcutaneous implants, studies have failed to support the *adhesive obturation of the root canal*, which consequently necessitates retreatment. 

According to Shipper and Trope [15], RS obtained better results with bacterial infiltration compared to GP. No difficulties have been reported for the removal of RS during retreatment procedures in comparison with GP [16]. 

Several studies evaluated the comparative efficacy of GP and RS regarding the duration of retreatment procedure and the presence of residual obturation material in the canal after retreatment. Therefore, the objectives of the present review were to* i*) identify the most efficient method for the retreatment of teeth obturated with RS and *ii*) to compare the efficacy of RS removal with GP in terms of the treatment duration and the presence of residual obturation material in the canal.

**Figure 1:**
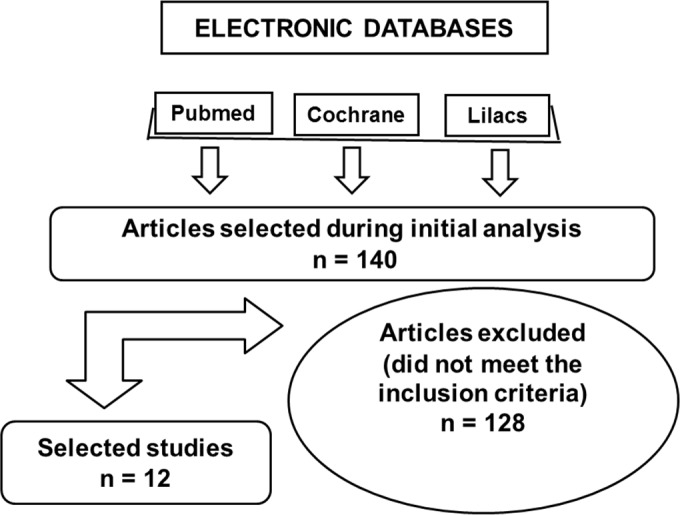
The search strategy

## Materials and Methods

***Literature search***

We conducted a systematic search in three international databases (PubMed, Cochrane Library and Lilacs) through the articles published from January 2001 to June 2014. Using the Boolean operators “AND” and “OR,” we used the following search terms: "Epiphany Sealer", "Resilon", "Retreatment", "Removal Procedure", and their corresponding synonyms, in varying combinations. The search strategies are shown in Table 1.

***Inclusion and exclusion criteria***

The sequence of steps in the literature search is illustrated in Figure 1. After analyzing the abstracts and the titles, three independent evaluators selected the relevant studies according to the inclusion and exclusion criteria established prior to the start of the research. Then, the selected papers were obtained and analyzed in full text. Finally, the selected articles were included in the data systematization process by the evaluators in a consensus meeting. 

We included longitudinal or cross-sectional randomized controlled studies and randomized *in vitro* studies because of the

small number of studies on this field. There were no limitations on the language or the date of publication. Literature reviews, case reports, unrelated studies and articles with questionable research sources or subjectivity were excluded. 

We also excluded studies not using the combination of RS and Epiphany cement because they were beyond the scope of this research. This study followed the guidelines of the Brazilian Cochrane collaboration.

***Summary measures and synthesis of results***

Initially according to the general characteristics, the obtained results were synthesized/organized in tables, after inclusion of those studies that compared the removal procedure of RS and GP.

## Results

After careful analysis and application of the inclusion and exclusion criteria, 13 articles were obtained for this review including three preliminary *in vitro* studies and nine randomized controlled clinical trials (as shown in Figure 1). 

The number of articles found in the databases is represented in Table 1. The total sample consisted of 512 teeth and 160 cones, plus 63 samples of obturation material. Table 2 summarizes the principal objective, results and conclusion of each study.

Table 3 shows the different techniques and their respective results. It is important not only to determine the most efficient technique but also to compare the data obtained with other filling materials.

## Discussion

The obturation materials must meet many criteria including sealing and biocompatibility; the material must also remain inert over time. However, in certain clinical situations such as endodontic retreatments, they should be easily removable and soluble with chemical solvents [16-20]. 

GP is the most commonly used obturation material and its excellent properties have made it the “gold standard” for root canal filling. However, removal of this material during retreatment is not always satisfactory, which can cause operative difficulties as well as biological problems [12, 21-23]. RS was introduced as an alternative filling material for root canals. Additionally, it is biocompatible and has improved adhesive ad sealing properties because of its polymeric nature. 

**Table 1:** Search strategies and number of articles found in the databases (PM=PubMed, CH=Cochrane, LL=Lilacs

**Search strategies**	**PM**	**CH**	**LL**
**(Epiphany sealer or Resilon) and (Retreatment or Removal Procedure)**	34	5	1
**(Epiphany sealer or Resilon) and (Retreatment or Residue Material)**	25	5	22
**(Epiphany sealer or Resilon) and (Retreatment or Solvents)**	34	6	4
**(Epiphany sealer or Resilon) and (Retreatment) and (Rotary NiTi Instruments)**	4	0	0

**Table 2:** Summary of the selected studies: objectives and key conclusions

**Title**	**Type of study**	**Objective of study**	**Sample**	**Key conclusions**
A Comparison of the Effectiveness of Chloroform in Dissolving Resilon and Gutta-Percha [24]	*In vitro* test	To evaluate the removal of RS/EP and GP/AH using chloroform as solvent	Not stated by the authors.	RS/EP had better solubility in chloroform than GP/AH.
Comparative Study of Removal of Current Endodontic Fillings [23]	Randomized trial	To evaluate the ease of removal of 4 obturation materials	72 teeth (G1-Resilon, G2-GuttaFlow, G3-Endotwinn, G4-gutta-percha).	There was no difference in the amount of residual material, but canals filled with GuttaFlow and EndoTwinn were removed more rapidly.
Comparison Between Gutta-Percha and Resilon Removal Using Two Different Techniques in Endodontic Retreatment [25]	Randomized trial	To compare the effectiveness of the removal gutta-percha/AH with two rotary systems (K3 and Liberator files)	80 teeth (G1-RS/EP; G2-GP/AH).	The RS/EP was removed faster than the GP/AH. The K3 system was the fastest in both groups.
Comparison between gutta-percha and resilon retreatment [22]	Randomized trial	To compare the amount of residual obturation material on the root canal walls filled with gutta-percha and resilon	30 teeth (G1-GP/AH; G2-RS/EP).	The RS/EP group had significantly more residual material in the canal than the gutta-percha group.
Dissolving efficacy of different organic solvents on gutta-percha and resilon root canal obturating materials at different immersion time intervals [20]	*In vitro* test	To compare and evaluate the dissolving effectiveness of various solvents used during endodontic retreatment on resilon and gutta-percha	160 cones no. 40 (G1 to G4-RS; G5 to G8-GP) Solvent: *1)* xylene; *2*) tetrachloroethylene; *3)* refined orange oil; and *4)* distilled water.	Xylene, orange oil, and refined tetrachloroethylene can be used to dissolve gutta-percha/Resilon during retreatment with various techniques. Xylene was the most efficient for all the groups.
Efficacy of 3 techniques in removing root canal filling material [18]	Randomized trial	To evaluate the effectiveness of three techniques for the removal of RS/EP and laterally compacted GP/AH in straight and curved canals	90 teeth (G1 to G3-canals filled with GP/AH; G4 to G6-canals obturated with RS/EP)	Removal of RS/EP resulted in less residual material and was faster than GP/AH in curved and straight canals.
Efficacy of retreatment techniques for a resin-based root canal obturation material [26, 27]	Randomized trial	To evaluate two retreatment techniques commonly used for removal of resilon (rotary systems in combination with heat or chloroform)	60 teeth (G1-RS/EP; G2-GP/AH)	Both techniques removed RS/EP faster than GP/AH. Chloroform in combination with the rotary systems was more efficient.
Efficacy of three different methods in the retreatment of root canals obturated with resilon/epiphany [26]	Randomized control trial	To evaluate the effectiveness of three techniques for the removal of the new RS/EP obturation system	30 teeth (G1-Mtwo R/Mtwo files; G2-Mtwo R and chloroform; G3-Mtwo R and Endosolv)	Endosolv R combined with rotary instruments was more efficient for the removal of the material when compared with chloroform.
Efficacy of two rotary NiTi instruments in removal of resilon obturants [17]	Randomized control trial	To evaluate the effectiveness of ProTaper and Mtwo-R in the removal of RS/EP, with or without the use of chloroform during retreatment	60 teeth (1) Mtwo R/solvent; (2) Mtwo-R; 3) ProTaper D/solvent; and 4) ProTaper D	RS/EP was removed more effectively from the apical third in the ProTaper/solvent group. Considering the whole canal, there were no differences between the groups.
Removal of resin-based root canal filling materials with K3 rotary instruments: relative efﬁcacy for different combinations of filling materials [28]	Randomized control trial	To compare the removal process time of the RS/EP system with the K3 system with or without heat softening using System B	40 teeth (G1-RS/EP; G2-Resilon+Super Bond; G3-GP+Super Bond; G4-Canals N+GP)	The filling material removal time using K3 was longer, but may be shortened when combined with System B.
Solvent capacity of different substances on gutta-percha and resilon [21]	*In vitro* test	To compare the effectiveness of three solvents (Xylol, eucalyptol and orange oil) for GP and resilon	21 specimens (G1-common GP; G2-thermoplastified GP; G3-Resilon)	All substances were efficient in dissolving the material, but Xylol was the most efficient.
Effectiveness of hand and rotary instrumentation for removing a new synthetic polymer-based root canal obturation material (epiphany) during retreatment [19]	Randomized trial	To compare the quantity of obturation material remaining in the root canal after manual and mechanized removal	60 teeth (G1-RS/EP, G2-GP/AH)	RS/EP was more effectively removed than GP/AH. The technique using Hedström instruments was faster than using RaCe instruments.

*GP/AH (gutta-percha in combination with AH Plus)* *,* *RS/EP (Resilon in combination with Epiphany cement*

However, the small number of clinical studies focusing on the longevity of canals obturated with RS casts some doubt on its long-term effectiveness [1, 22-24].

The data obtained from different studies are rather new and thus need to be systematized and validated before being used in evidence-based decision-making. 

The first difficulty in this study stemmed from the relative lack of clinical research. This suggests that more clinical researches must be conducted to clarify the advantages and disadvantages of the use of new obturation systems as well as their impact during endodontic retreatment. Hence, the inclusion criteria were somewhat flexible.

Cleaning of the root canals during retreatment is extremely important because it is necessary to control the infection that perpetuated or initiated the process of periapical damage to the periradicular tissues in most cases. The amount of debris remaining in the root canals after removal of the obturation material can be considered as a negative factor; therefore, this review focuses mainly on evaluating the residual debris. The duration of the procedure was a secondary factor in the evaluation [16].

Azar *et al.* [24] assessed the solubility of obturation materials in chloroform which is widely used for removal of the root obturation material. Higher solubility of RS system was observed in comparison with GP and AH-Plus sealer. 

There is some evidence that removal of RS is more efficient than GP during retreatment; four out of six studies comparing these obturation materials obtained better results with RS [18, 19, 25, 27]. The work by Taşdemir *et al. *[23] claimed that GuttaFlow is more efficient than RS and GP, whereas Zarei *et al.* [22] found conflicting data that allowed them to state that there was a significant difference between RS and GP. However, the latter study was limited to a visual evaluation using photographs, which could have led to observational errors.

Two *in vitro* studies [20, 21] have evaluated different chemical substances and their capacity in dissolving RS and GP cones. The data are consistent in concluding that Xylol is more efficient among the two other evaluated solvents and RS is more soluble than GP. This can be considered a positive factor in the removal of RS cones and making the technical process shorter. Moreover, even though Xylol is more efficient, RS is quite soluble in other chemical products such as orange oil, tetrachloroethylene and eucalyptol, making it more advantageous. 

Three studies compared the mechanical techniques with and without different chemicals in removal of debris from canals filled using RS. According to the first study, Endosolv is more efficient than chloroform for removing the root filling because the procedure is faster and less debris is left behind. In all the groups, the material was removed using the Mtwo R system [26]. In another study, Desadresanfar *et al.* [17] showed that the ProTaper system removes RS more efficiently than the Mtwo R system. In an evaluation of RS removal using K3 system with and without System B (physical technique-heat), Iizuka *et al.* [28] observed that the procedure time was longer with the standalone K3 system. In addition, they observed that the technique was more efficient when used in combination with System B. Therefore, this technique is indicated for removing RS from the obturated canals. 

The limitation of our study is that it was not a systematized review with meta-analysis. This can be attributed to the relative scarcity of the literature discussing retreatment methods after treatment with relatively new filling materials. New materials need standardized studies on their clinical effectiveness. For a more thorough analysis, greater standardization of the methodologies is required to evaluate the effectiveness of RS.

**Table 3:** Studies that compare the removal of RS/EP and GP/AH by mechanical and chemical techniques

**Study**	**Mechanical technique**	**Chemical technique**	**Most efficient**
Comparative study of removal of current endodontic fillings [23]	Mtwo-R instruments and Mtwo instruments	Chloroform	RS/EP and GP/AH were less efficient that GuttaFlow
Comparison between gutta-percha and resilon removal using two different techniques in endodontic retreatment [25]	K3 System and Liberator files	Sodium hypochlorite and EDTA	RS/EP was removed faster than GP/AH. The K3 system was more efficient
Comparison between gutta-percha and resilon retreatment [22]	Gates-Glidden drills	Chloroform	There was more debris in the RS/EP than in the GP/AH group
Efficacy of 3 techniques in removing root canal filling material [18]	Gates-Glidden drills with or without chloroform system B	Chloroform	RS/EP was removed faster and left less debris
Efficacy of retreatment techniques for a resin-based root canal obturation material [27]	ProFile System B	Chloroform	RS/EP was removed faster. The best technique was the association between rotary systems and chloroform
Effectiveness of hand and rotary instrumentation for removing a new synthetic polymer-based root canal obturation material (epiphany) during retreatment [19]	RaCe rotary Hedström ﬁles	Not used	RS/EP was removed more efficiently than GP/AH. The Hedström file technique was faster than the Race instruments

## Conclusion

The results of this review suggest that the ProTaper system is the most efficient method for removing Resilon; in addition, the most efficient technique seems to be a combination of manual and rotary instruments with chemical solvents. The results also indicate that Resilon is easy to remove and has similar or superior solubility in chemical solvents than gutta-percha, although it may result in more remnants of debris.

## References

[1] de Souza Filho FJ, Gallina G, Gallottini L, Russo R, Cumbo EM (2012). Innovations in endodontic filling materials: guttapercha vs Resilon. Curr Pharm Des.

[2] Lee B-S, Wang C-Y, Fang Y-Y, Hsieh K-H, Lin C-P (2011). A Novel Urethane Acrylate-based Root Canal Sealer with Improved Degree of Conversion, Cytotoxicity, Bond Strengths, Solubility, and Dimensional Stability. J Endod.

[3] Ashraf H, Samiee M, Eslami G, Ghodse Hosseini MR (2007). Presence of Candida Albicans in Root Canal System of Teeth Requiring Endodontic Retreatment with and without Periapical Lesions. Iran Endod J.

[4] Sunde PT, Olsen I, Debelian GJ, Tronstad L (2002). Microbiota of periapical lesions refractory to endodontic therapy. J Endod.

[5] Siqueira JF (2001). Aetiology of root canal treatment failure: why well-treated teeth can fail. Int Endod J.

[6] Anderson AC, Hellwig E, Vespermann R, Wittmer A, Schmid M, Karygianni L, Al-Ahmad A (2012). Comprehensive analysis of secondary dental root canal infections: a combination of culture and culture-independent approaches reveals new insights. PLoS One.

[7] Lin LM, Skribner JE, Gaengler P (1992). Factors associated with endodontic treatment failures. J Endod.

[8] Stabholz A, Friedman S (1988). Endodontic retreatment--case selection and technique Part 2: Treatment planning for retreatment. J Endod.

[9] Cunha RS, De Martin AS, Barros PP, da Silva FM, Jacinto RC, Bueno CE (2007). In vitro evaluation of the cleansing working time and analysis of the amount of gutta-percha or Resilon remnants in the root canal walls after instrumentation for endodontic retreatment. J Endod.

[10] Wilcox LR, Krell KV, Madison S, Rittman B (1987). Endodontic retreatment: evaluation of gutta-percha and sealer removal and canal reinstrumentation. J Endod.

[11] Friedman CE, Sandrik JL, Heuer MA, Rapp GW (1977). Composition and physical properties of gutta-percha endodontic filling materials. J Endod.

[12] Miner MR, Berzins DW, Bahcall JK (2006). A comparison of thermal properties between gutta-percha and a synthetic polymer based root canal filling material (Resilon). J Endod.

[13] Teixeira FB, Teixeira EC, Thompson J, Leinfelder KF, Trope M (2004). Dentinal bonding reaches the root canal system. J Esthet Restor Dent.

[14] Versiani MA, Carvalho-Junior JR, Padilha MI, Lacey S, Pascon EA, Sousa-Neto MD (2006). A comparative study of physicochemical properties of AH Plus and Epiphany root canal sealants. Int Endod J.

[15] Shipper G, Trope M (2004). In vitro microbial leakage of endodontically treated teeth using new and standard obturation techniques. J Endod.

[16] Somma F, Cammarota G, Plotino G, Grande NM, Pameijer CH (2008). The effectiveness of manual and mechanical instrumentation for the retreatment of three different root canal filling materials. J Endod.

[17] Dadresanfar B, Iranmanesh M, Mohebbi P, Mehrvarzfar P, Vatanpour M (2012). Efficacy of Two Rotary NiTi Instruments in Removal of Resilon/Epiphany Obturants. Iran Endod J.

[18] Bodrumlu E, Uzun O, Topuz O, Semiz M (2008). Efficacy of 3 techniques in removing root canal filling material. J Can Dent Assoc.

[19] Schirrmeister JF, Meyer KM, Hermanns P, Altenburger MJ, Wrbas KT (2006). Effectiveness of hand and rotary instrumentation for removing a new synthetic polymer-based root canal obturation material (Epiphany) during retreatment. Int Endod J.

[20] Mushtaq M, Farooq R, Ibrahim M, Khan FY (2012). Dissolving efficacy of different organic solvents on gutta-percha and resilon root canal obturating materials at different immersion time intervals. J Conserv Dent.

[21] Tanomaru-Filho M, Orlando T, Bortoluzzi EA, Silva GF, Tanomaru JM (2010). Solvent capacity of different substances on gutta-percha and Resilon. Braz Dent J.

[22] Zarei M, Shahrami F, Vatanpour M (2009). Comparison between gutta-percha and Resilon retreatment. J Oral Sci.

[23] Taşdemir T, Yildirim T, Celik D (2008). Comparative study of removal of current endodontic fillings. J Endod.

[24] Azar M, Khojastehpour L, Iranpour N (2011). A comparison of the effectiveness of chloroform in dissolving resilon and gutta-percha. J Dent (Tehran).

[25] de Oliveira DP, Barbizam JV, Trope M, Teixeira FB (2006). Comparison between gutta-percha and resilon removal using two different techniques in endodontic retreatment. J Endod.

[26] Ramzi H, Shokouhinejad N, Saghiri MA, Samieefard A (2010). Efficacy of Three Different Methods in the Retreatment of Root Canals Filled with Resilon/Epiphany SE. Iran Endod J.

[27] Ezzie E, Fleury A, Solomon E, Spears R, He J (2006). Efficacy of retreatment techniques for a resin-based root canal obturation material. J Endod.

[28] Iizuka N, Takenaka S, Shigetani Y, Okiji T (2008). Removal of resin-based root canal filling materials with K3 rotary instruments: relative efficacy for different combinations of filling materials. Dent Mater J.

